# Utility of knife-edge position tracking in cycloidal computed tomography

**DOI:** 10.1364/OE.470798

**Published:** 2022-11-09

**Authors:** Oriol Roche i Morgó, Jure Aleksejev, Alberto Astolfo, Savvas Savvidis, Mattia FM Gerli, Silvia Cipiccia, Alessandro Olivo, Charlotte K. Hagen

**Affiliations:** 1 Dept. of Medical Physics and Biomedical Engineering, University College London, WC1E 6BT, UK; 2 UCL Divison of Surgery and Interventional Science, University College London, Rowland Hill Street, London, NW3 2P, UK

## Abstract

Cycloidal computed tomography provides high-resolution images within relatively short scan times by combining beam modulation with dedicated under-sampling. However, implementing the technique relies on accurate knowledge of the sample’s motion, particularly in the case of continuous scans, which is often unavailable due to hardware or software limitations. We have developed an easy-to-implement position tracking technique using a sharp edge, which can provide reliable information about the trajectory of the sample and thus improve the reconstruction process. Furthermore, this approach also enables the development of other innovative sampling schemes, which may otherwise be difficult to implement.

## 1. Introduction

**Fig. 1.**
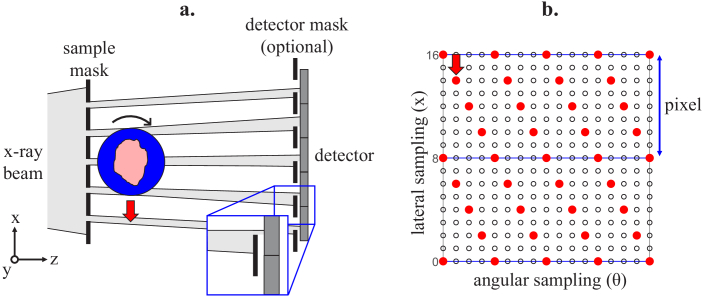
(a) Schematic of a cycloidal CT setup, including an optional detector mask which can provide access to a phase contrast channel. The inset at the bottom illustrates the phase contrast principle – by adding the detector mask, refraction of the beamlets translates into a change in intensity on the detector pixel. The sample rotates and translates at the same time, which creates the interlaced data spread represented on (b). The red dots represent acquired datapoints, while the empty dots represent the data that would also be acquired in a dithered scan. The blue lines show the boundaries between pixels (or periods, accounting for magnification).

Cycloidal computed tomography (CT) is a recently developed micro-CT imaging technique which provides high-resolution images efficiently through the combination of a highly structured x-ray beam and innovative sampling schemes [1,2]. Usually, spatial resolution in x-ray imaging is limited by the combined blur of the source point spread function (PSF) and the detector pixel response function [3]; however, higher spatial frequencies can be accessed by dividing the x-ray beam into an array of beamlets (see Fig. 1(a)) with the use of an absorbing mask. The exact structure of the beamlets is determined by the width w of the slit-shaped apertures of the mask, which are periodically separated by a distance p. The period p of the mask usually matches the size of the detector pixels (a), accounting for magnification. To first approximation, the usage of such a mask provides access to spatial frequencies on the order of 1/w [4,5], if the following conditions are fulfilled: firstly, w must be smaller than the projected source width scaled to the sample plane. Secondly, the beamlets must remain reasonably well separated. Finally, the PSF of the detector must be sufficiently narrow that it does not cause significant blurring post detection. In case the latter requirement is not met, it is possible to use “skipped” masks, which have a larger period, matching the size of two or more pixels (i.e., p = m·a, where m = 1, 2, 3…), to increase the distances between the beamlets. The use of photon-counting detectors, which generally provide a narrower PSF, is also an option.

However, the use of a mask imposes an under-sampling problem: if the sample is simply rotated inside the structured beam (a “rotation-only” scan), there are large areas which remain unseen behind the opaque regions of the mask. Hence, signals are sampled well below the Nyquist criterion, limiting the spatial resolution of the acquired image despite the presence of higher frequencies, and potentially causing under-sampling artefacts. In cycloidal CT, this is alleviated by rotating and simultaneously translating the sample in subpixel steps along the scanner’s x-direction (“roto-translation”). While this does not remove the under-sampling, since the number of collected datapoints does not change with respect to a rotation-only scan, the acquired data become more evenly spread across the sinogram (Fig. 1(b)), which facilitates the achievement of a higher in-slice resolution using a suitable data recovery method (e.g., bivariate interpolation) [2].

Under-sampling in CT is a common field of study, typically aimed at improving its (dose or scan time-related) efficiency [6–11]. Cycloidal CT indeed shares similarities with studies on under-sampling in the projection space [12–14], but also differs, not least because it offers access to phase contrast, which can provide a higher contrast-to-noise ratio for weakly attenuating samples than attenuation contrast. The phase contrast implementation requires the addition of a detector mask which makes the system sensitive to refraction (further details in section 2.2) [15], or the use of a detector with pixels smaller than the beamlets [16].

Previously, the under-sampling imposed by a mask as shown in Fig. 1(a) was handled by applying a procedure called “dithering”. This method involves scanning the sample in several subpixel steps at each rotation angle until it has been fully illuminated (i.e., the beamlets have covered one mask period in multiple steps); the sample is then rotated to acquire the next projection, where this process is repeated, and so on [4,5]. This process has similarities with super-resolution imaging, in which several low-resolution frames at different positions are combined to produce a high-resolution equivalent [17,18]; in our case, the subpixel shifting is done to alleviate the under-sampling caused by the use of absorption masks. While dithering ensures that high spatial frequencies are properly captured, providing a high in-slice resolution in the reconstructed images, it also requires the rotation motor to stop at every projection, which increases scan time, reduces efficiency, and is therefore unsuitable for applications which require a high sample throughput.

Cycloidal CT can provide similarly high in-slice resolutions, with the added advantage that scans can be performed continuously (in “flyscan” mode), which eliminates overheads and thus reduces scanning time to the actual exposure time. The precise gain in speed depends on the setup and acquisition parameters; in the specific case discussed in this paper, the continuous cycloidal scans were approximately 40 times shorter than the dithered scans. This can be useful in many applications, particularly those with tight time constraints, such as intraoperative imaging [19,20].

Cycloidal flyscans can be implemented unidirectionally, where the sample moves from one side of the field-of-view (FOV) towards the other during its rotation; as a back-and-forth movement, where the sample travels between two points repeatedly; or as a “pixelwise” scan, where the back-and-forth movement is limited to within a pixel-size equivalent distance [21]. Pixelwise scanning has the advantage that a FOV only slightly larger than the object is sufficient, while in the other implementation strategies the FOV must be larger than the sample to accommodate the extended motion along the x-direction. In all cases, a “regridding” step is necessary, through which the data are placed into their correct sub-pixel position in the sinogram space. In the cases where the sample movement extends beyond one (de-magnified) pixel, this involves shifting the data by the number of pixels the sample has covered before allocating them to their correct sub-pixel position.

Because of this regridding step, all implementations share the need for accurate knowledge of the sample’s subpixel position (along x) during a scan. Earlier work [1,2,21] assumed correctness of the nominal sample trajectory, however in practice a variety of factors such as drifts, vibrations or motor inaccuracies mean that the real sample trajectory may differ, which can compromise the regridding process; this applies to the pixelwise implementation in particular. This, in turn, has a detrimental effect on image quality and resolution. These issues can be circumvented by manual inspection of each acquired frame in order to estimate the subpixel position of the sample, but this approach is time-consuming (upwards of 1000 frames are often acquired) and unreliable, which defeats the aforementioned benefits of cycloidal CT. For simple samples (e.g., those with a perfectly cylindrical sample holder), the regridding could also be done by ensuring a smooth boundary of the sinogram; however, this is difficult to implement for complex or low-contrast samples.

To solve this issue, we have developed an easy-to-implement position tracking method based on tracking a knife edge, which exhibits subpixel accuracy and thus informs on the exact sample trajectory during a scan. Thanks to this information, accurate tomographic reconstructions can be obtained in a standardized manner, even when the nominal and real motions of the sample during a cycloidal CT scan differ.

In this paper, we discuss the merits of applying knife-edge sample tracking in cycloidal CT. First, we show its ability to track subpixel sample movements. We then demonstrate its role in the reconstruction of accurate tomographic images with cycloidal CT. Finally, moving beyond the use of knife-edge tracking as a corrective measure, we discuss its role as a facilitator for the development of new sampling schemes (demonstrated on the extreme case of a random-walk CT scan), for which an accurate knowledge of the sample position is paramount.

## 2. Material and methods

### 2.1 Knife-edge position tracking

**Fig. 2.**
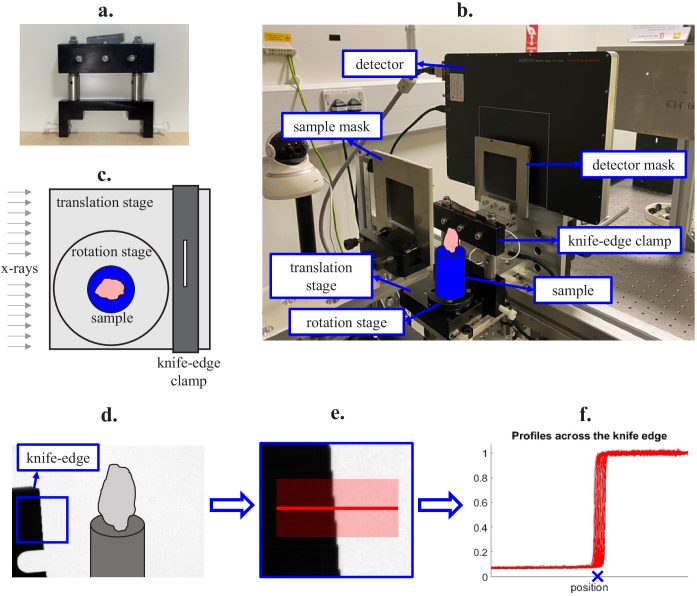
The experimental setup for knife-edge tracking is presented in panels (a) to (c). We used a purpose-built clamp to keep the scraper blade in place (a), which could then be tightly affixed to the sample motor (b). As can be seen in the schematic in (c), the clamp was attached to the translation stage and not the rotation stage, allowing us to separate the two movements. Panel (d) illustrates the position of the knife edge relative to the sample. Panels (e) and (f) illustrate the process of extracting the edge position from a frame: profiles are extracted across several rows, the x-position of each profile’s midpoint is found, and the average x-position is used to estimate the edge position, marked with an X in panel (f).

Knife-edge position tracking requires a sharp object mounted on a stable clamping system. The object must be highly attenuating in order to obtain a clear edge profile. Sharper edges may provide higher accuracy results, but in practice the required accuracy is determined by the resolution capabilities of the scanner. The stability of the blade is crucial so that its motion represents, accurately, the motion of the sample. To isolate the lateral movement, the rotation stage must be above the sample translation stage, and the clamp must be affixed onto the latter but not the former (see Fig. 2). Sub-pixel motion can be tracked by tilting the edge of the blade. The method appears to perform well at a wide range of tilt angles, as long as the blade is not perpendicular or parallel to the direction of translation.

The edge position is estimated by extracting a profile across one row of pixels across the edge of the tilted blade. The profile is interpolated onto a finer grid to obtain subpixel accuracy. The position of the edge is approximated as the x-position that corresponds to the mean between the (averaged) intensities on the left and right sides of the edge. Fitting an error function can be advantageous if the edge signal is noisy. In our case, we found that repeating the above process across several pixel rows and averaging the results provided sufficiently robust position estimates.

The positions are obtained in pixel units and can be used to correctly position the acquired projection data in the sinogram. Distances measured at the detector plane need to be scaled to the sample plane by accounting for magnification.

### 2.2 Edge-illumination x-ray phase contrast imaging

X-Ray Phase Contrast imaging (XPCi) exploits the changes in the phase of x-rays when they travel through a sample to drive image contrast, alongside the conventional attenuation. For weakly attenuating materials, such as biological tissue, this can provide increased contrast [22,23].

An experimental setup as described above (Fig. 1(a)) can be turned into an Edge Illumination (EI) XPCi system by adding a second mask, often called a “detector mask”, in front of the detector [15,24,25]. Like the absorbing “sample” mask, it has periodically spaced slit-shaped apertures, the period of which matches that of the sample mask accounting for the magnification of the system. The two masks are aligned so that, without a sample, approximately half of each beamlet falls on the exposed detector pixel and the other half on the absorbing septa of the mask. With the introduction of a sample, the occurring refraction leads to small changes to the x-rays’ direction of propagation, which will cause a beamlet to fall further into the pixel or further into the septum. This, in turn, leads to a change in the intensity recorded by the detector, which gives rise to contrast. A more in-depth introduction to EI XPCi is available in [15].

For the reconstruction of tomographic images from those data, a phase retrieval procedure must first be applied to establish a suitable line integral relationship between the data and the object function to be reconstructed. In this paper, we have used a “single-shot” approach [26,27], whereby it is sufficient to acquire a single frame for every position of the sample. The single-shot retrieval assumes a homogeneous sample, although in practice it has been found to be applicable for samples composed of similar materials (e.g., soft tissue). In cycloidal acquisitions, phase retrieval is performed after the data recovery step.

EI XPCi shares similarities with other XPCi methods such as grating interferometry (GI) [28–30], which captures changes in the differential of the phase. Like EI, the latter can be implemented with table-top x-ray sources thanks to its relaxed coherence requirements.

### 2.3 Experimental setup

In our experiments, we used a scraper blade held in place by a clamp (Fig. 2) placed on the translation stage, immediately downstream of the sample.

All data were acquired with the system in phase contrast mode, i.e., using an EI setup (Fig. 1(a)). This allowed us to demonstrate our method on weakly attenuating samples, including biological tissue. We used a Hamamatsu CMOS-based flat panel C9732DK-11 detector, with 50 × 50 µm pixels, and a Rigaku MicroMax 007 HF x-ray source with a Molybdenum target operating at 40 kVp and 25 mA. The source and detector were separated by 0.875 m, with the sample mask 0.7 m downstream from the source and 0.037 m before the sample. The sample mask had 10 µm apertures and a 79 µm period, while the detector mask had 17 µm apertures and a 98 µm period. Hence, every other detector pixel was “skipped” to reduce the adverse effects of crosstalk between pixels. Consequently, the effective pixel size at the sample stage was 83 µm.

The raw data were corrected using flat fields to account for background inhomogeneities, and dark fields for detector noise correction. Cycloidal images were reconstructed using the knife-edge method unless stated otherwise; specifically, the results from the tracking procedure described above were used to accurately position the acquired data in the sinogram space. The missing sinogram entries were then filled using bicubic splines interpolation, and tomographic reconstruction was performed with MATLAB’s implementation of filtered back-projection. Although our system utilises a cone beam, the assumption of a parallel beam geometry was made, justified by small sample sizes and the relatively large distance between source and sample.

## 3. Results

### 3.1 Tracked versus nominal motion

**Fig. 3.**
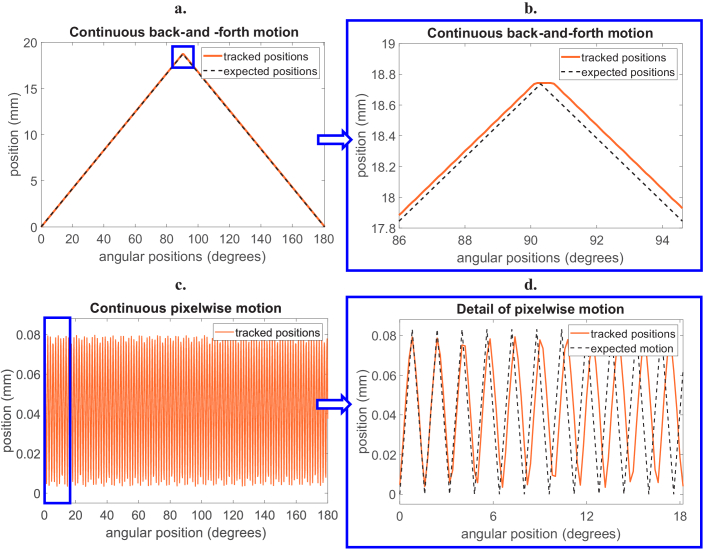
Comparison of sample motion based on knife-edge position tracking results, in orange, and the expected nominal motion in black, for a cycloidal back-and-forth flyscan (a-b) and a cycloidal pixelwise flyscan (c-d).

Knife-edge position tracking was applied to two types of cycloidal flyscans: continuous back-and-forth and continuous pixelwise scans. In both cases, the reconstruction of images had so far been challenging due to the absence of accurate motion profiles. Here, we compare the tracked and nominal sample translations in the two scenarios. For the continuous back-and-forth scan (Fig. 3(a)-(b)), although the expected and nominal motions are close, they are not completely overlapping, which worsens when the sample changes the direction. For the continuous pixelwise cycloidal scan, which is arguably the most challenging implementation of cycloidal CT, a much greater discrepancy between tracked and nominal motions can be seen (Fig. 3(c)-(d)). Specifically, it becomes apparent that the sample changes its direction slightly before reaching the end of one mask period (0.083 mm), which creates a lag that gets increasingly severe as the scan progresses.

**Fig. 4.**
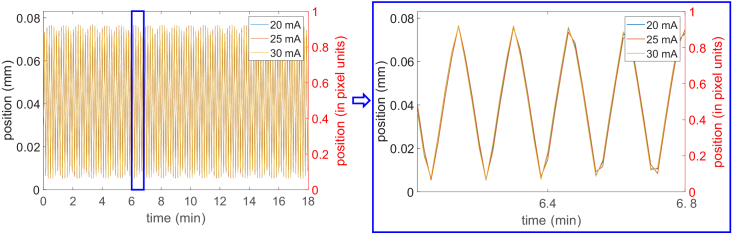
Tracking of a pixelwise scan, using the knife edge, at three different currents (20 mA, 25 mA, 30 mA). The results suggest that the output of knife-edge tracking remains similar at increasing noise level.

Before using the tracked motion profiles to reconstruct tomographic images, we wanted to understand whether the tracking method had a dependency on the noise level in the images; if that were the case, positions tracked at low tube current would have larger errors than those tracked at high current, which would be undesirable, as it would effectively limit accurate tracking to high-dose scans. For this purpose, we performed three pixelwise scans with increasing x-ray tube currents, and compared the tracked positions. The results (Fig. 4) show that the tracking output does not significantly change with current.

### 3.2 Usage in cycloidal CT

**Fig. 5.**
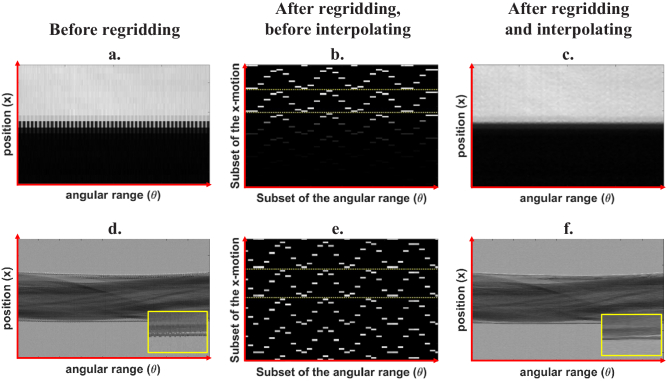
The process of regridding and interpolating with pixelwise cycloidal data is illustrated in the images above. Panels (a) to (c) represent the process for the knife edge “pseudo-sinogram”. The flatness of the edge can be used as a metric for accurate regridding and interpolation. Panel (b) details the regridding and interpolation of the sinogram with the sample; all acquired datapoints are placed in their corresponding sub-pixel “slots” on the sinogram according to the positional information obtained from the knife-edge tracking. The dotted yellow lines in the middle images represent the edges of the pixel (or the mask period, accounting for magnification). The same is shown in panels (d) to (f), but this time for a real sample (a preserved rat heart in a plastic cylinder).

As explained above, the knife-edge tracking method was developed to improve the regridding process that is a core part of cycloidal CT. Based on the tracked motion profiles, the regridding can be automated, with no additional input required from the user. A metric to assess whether the sinogram has been accurately regridded is provided by the images of the knife edge. By extracting profiles from each frame in which the knife edge is in a different position and assembling these into a separate matrix, a “pseudo-sinogram” is created (Fig. 5). After applying the same regridding and interpolation procedures that were applied to the “real” (i.e., sample) sinograms, the “pseudo-sinogram” should show a flat edge across all frames. Should this not be the case, any discrepancy should be corrected, and the same correction applied to the sample sinogram.

We applied knife-edge tracking to cycloidal CT scans of two different samples: a custom phantom made of polyethylene spheres and a preserved 300 g Sprague-Dawley rat heart. The polyethylene phantom contained spheres between 425 and 500 µm in diameter in a 3 mm plastic straw. The heart sample was obtained from the UCL BSU, from rats euthanised for organ harvesting via Schedule 1 methods. The specimen was fixed (using 4% paraformaldehyde solution) over a 24 h period and dehydrated using “critically point drying” as per Savvidis et al [31]. The heart was kept in a 10 mm 3D-printed sample holder during imaging.

For the sphere phantom, data were acquired over 180° with angular steps of 0.2°, corresponding to a total of 900 frames, at an exposure time of 1.2 s per frame. For the rat heart sample, 1800 frames were acquired over 180° with 1.2 s of exposure time, corresponding to an angular step of 0.1° (a smaller angular step was chosen to account for the greater sample diameter). Cycloidal scans were implemented by translating the sample along the x-direction by a nominal distance of 0.0208 mm (which corresponds to ¼ of the mask period when adjusted for magnification) per each angular step. For the sphere phantom, a continuous unidirectional approach was applied, whereby the sample moved by a total distance of 18.7135 mm from one side of the FOV to the other. For the rat heart, a continuous back-and-forth motion was applied, made necessary by the fact that the sample had a larger diameter and would thus have exited the FOV if moved unidirectionally. Specifically, the rat sample travelled by 18.7135 mm before changing direction and travelling back by the same distance. Continuous pixelwise scans were also performed for both samples, during which the samples were “wiggled” between the edges of a de-magnified pixel, which corresponds to a total distance of 0.083 mm, acquiring frames at (nominally) every ¼ of that distance.

For both samples, dithered scans, acquired with 16 dithering steps, were performed for reference. Rotation-only scans were mimicked by sub-sampling the dithered data (i.e., discarding all but the first dithering step acquired), then applying the same processing as for the cycloidal scans, bar the regridding.

**Fig. 6.**
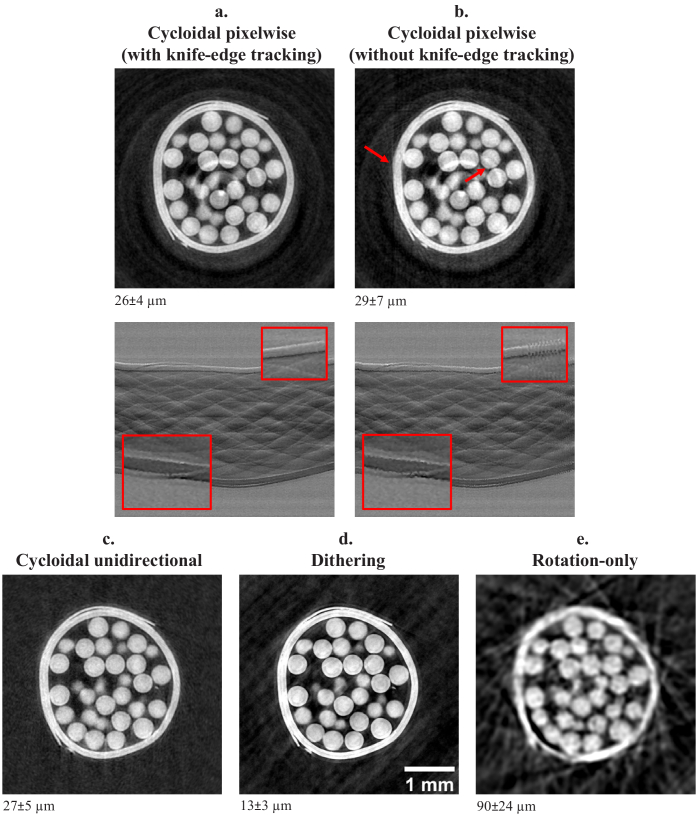
Reconstructed images of a polyethylene spheres phantom using different strategies, including cycloidal pixelwise scanning (a, b), cycloidal unidirectional scanning (c), dithering, which is taken as the gold standard (d), and rotation-only (e). Panels (a) and (c) have been reconstructed with the positional information extracted using knife-edge tracking. Panel (b) shows the cycloidal pixelwise image, reconstructed via visual inspection of the frames instead of knife-edge tracking. The red arrows point at artefacts created with the manual method. Sinograms are shown for panels (a) and (b) to illustrate the differences in results when using knife-edge tracking vs. manual alignment. The spatial resolution measurements for each slice can be found at the bottom left of each image.

**Fig. 7.**
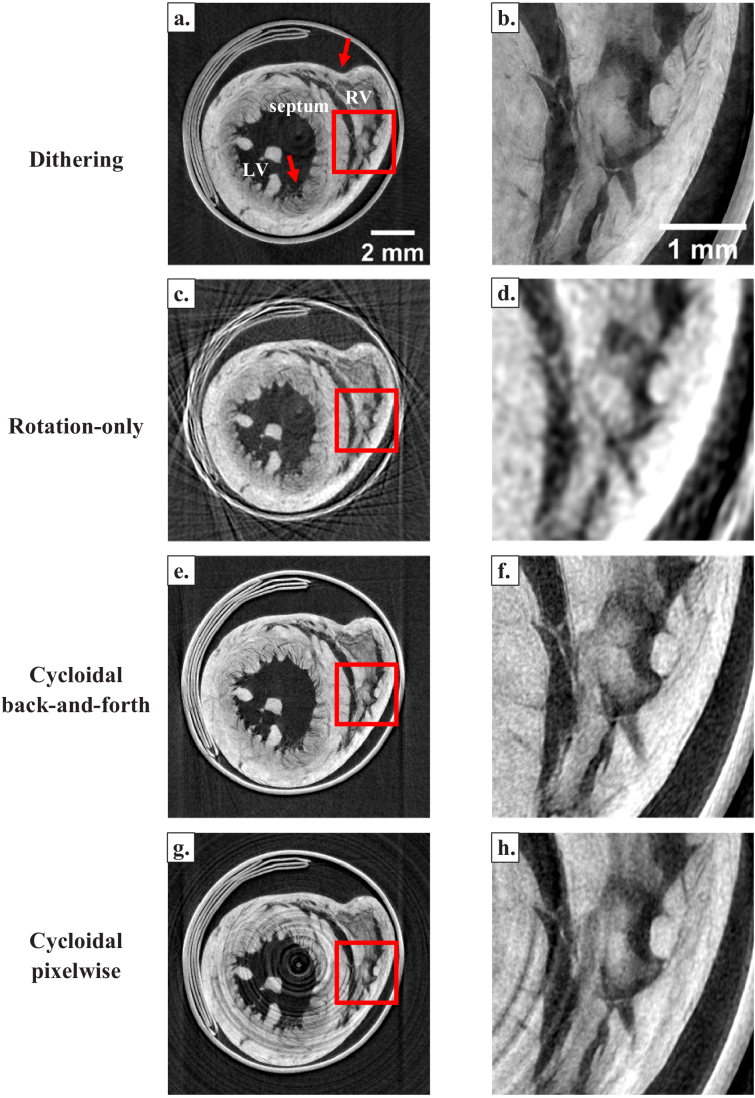
Reconstructed images of a rat heart using different strategies, including dithering (a, b), which is considered the gold standard, rotation-only scanning (c, d), cycloidal continuous back-and-forth scanning (e, f) and cycloidal pixelwise scanning (g, h). The latter two have been reconstructed using the positional information extracted with knife-edge tracking. On the right column, it can be appreciated that cycloidal strategies preserve most of the detail visible in the dithered image, although noise is somewhat higher. On panel (a), “LV” refers to “left ventricle” and “RV” refers to “right ventricle”, and the septum is the wall separating the two. The arrow at the top of the image points at a dent which must have been inflicted during the drying process. The arrow at the bottom indicates the striations of the cardiac muscles.

The results are shown in Fig. 6 and 7. The superiority of cycloidal CT over a rotation-only scan can be noted for both samples. Indeed, the quality of the cycloidal images is comparable to the dithered ones. This is despite the cycloidal images being reconstructed from the same number of frames as the rotation-only ones, and from 16 times fewer frames than the dithered ones. While the relative performance of the different scanning schemes had already been examined [21], here we analyse their overall quality in order to determine the benefits of applying knife-edge tracking.

The effect of knife-edge tracking can be observed by comparing the continuous pixelwise image reconstructed with (Fig. 6(a)) and without it (Fig. 6(b)). To obtain the latter image, the pixelwise image was reconstructed following a manual alignment of the acquired frames. Qualitatively, both images are similar. On closer inspection, however, it can be seen that the latter is affected by artefacts particularly around the edges of the container (see red arrows in Fig. 6(b)). This can also be seen in the corresponding sinogram, where there is misalignment for at least several views.

We measured the spatial resolution of the images for quantitative comparison. Resolution was estimated by extracting a line profile across the spheres and the container at 18 different positions in the slice, fitting an error function, calculating the corresponding derivative, and finding the full-width half-maximum of the resulting curve; all obtained values were then averaged. We found that knife-edge tracking provides 26 ± 4 µm, while for the manual processing method the resolution was 29 ± 7 µm. In a previous paper, we reported a spatial resolution of around 50 µm with manual processing [21], which highlights the lack of consistency of this approach. The spatial resolution of the cycloidal continuous unidirectional image (also reconstructed with knife-edge tracking) was estimated at 27 ± 5 µm.

Overall, these results suggest that knife-edge tracking provides a reliable alternative to manual regridding, maintaining and even improving the quality of acquired images, while making the reconstruction process itself fully automatable.

As can be seen in Fig. 6(a), one downside of the pixelwise approach is that it is prone to ring artefacts, which arise from local variations in the mask or in the performance of one or more detector pixels. Ring artefacts may be corrected by dedicated algorithms [32,33], but another option would be to expand the sample translation to the width of several pixels, rather than one. In this way, local variations in the mask and/or pixels will no longer affect the same lateral position of the scan, hence stripes (i.e., the manifestation of ring artefacts in the sinogram) will be broken up, eventually leading to the artefacts averaging out during the tomographic reconstruction. While such an acquisition may be considered a back-and-forth cycloidal scan, rather than a pixelwise one, it should be noted that by limiting the sample translation to only a few pixels the FOV requirements will only change minimally.

The images of the rat heart support the observations made for the sphere phantom, in that the knife-edge tracking method enables high quality cycloidal reconstructions, which outperform the rotation-only approach. The rat heart displays finer details than the plastic sphere phantom, which are largely absent in the rotation-only image (Fig. 7(c)). In Fig. 7(a), we can clearly see the muscle striations on the walls of the left ventricle and the septum, as well as the material inside the right ventricle, which remained in that position possibly because of the visible dent on the heart pushing it towards the centre of the ventricle (top arrow). Despite featuring the same amount of data as the rotation-only case, the cycloidal approaches retain a much greater amount of detail, as seen on the continuous back-and-forth (Fig. 7(e)) and continuous pixelwise images (Fig. 7(g)). The continuous pixelwise image again shows ring artefacts, obscuring some detail, although most features of the heart can still be appreciated.

### 3.3 Utilising knife-edge tracking as an enabler for the development of new scanning schemes

The ease of implementation of knife-edge tracking, and the promising performance on cycloidal CT data, suggest it can be used to explore other sampling strategies, related or unrelated to cycloidal CT, which require accurate knowledge on the sample position during scans. To demonstrate that the knife-edge method can track arbitrary motion profiles, we have implemented (and tracked) a “random-walk” CT scan, whereby the sample is translated along the scanner’s x-direction, but its direction of travel is changed pseudo-randomly (i.e., a random vector of “turnarounds” was generated and used to drive the sample translation stage) as the scan progresses. In this scenario, the regridding process crucially relies on the availability of accurate motion profiles. A random-walk CT scan may be considered an “extreme” case of a sample motion that is challenging to track, which is why it has been chosen as an example in the context of this paper. Furthermore, like the pixelwise approach, it has the added benefit that minimal demands on the FOV can be maintained (i.e., the scan can be set up so that the sample only moves within a short distance, e.g., a few pixels, ensuring that it will not exit the FOV at any point) while the issue of ring artefacts is alleviated, as per the explanation given above.

**Fig. 8.**
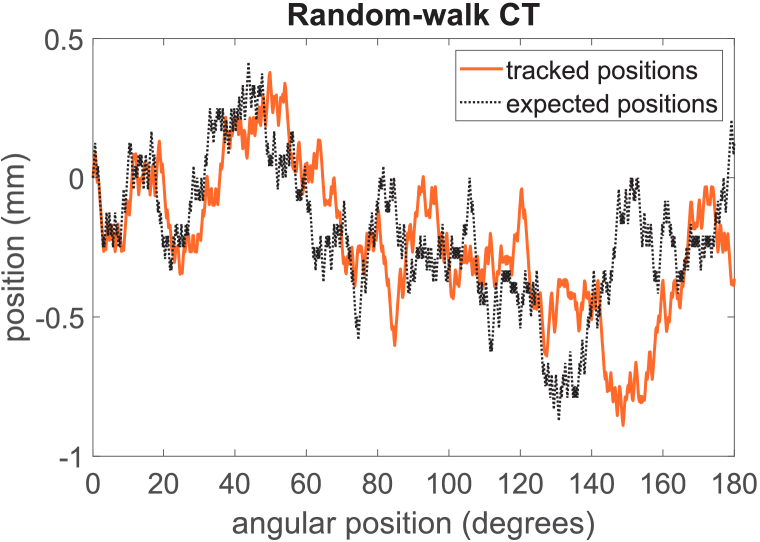
Tracked sample translation motor positions (in orange) for a random-walk scan, compared with the expected position (in black). The motor motion is progressively delayed as the scan goes on.

Figure 8 shows the comparison between the expected and tracked motion for the random-walk scan. As can be seen, the experimental results do not match the nominal positions. As in the pixelwise scan, there appears to be a lag in the sample movement which accumulates throughout the scan.

**Fig. 9.**
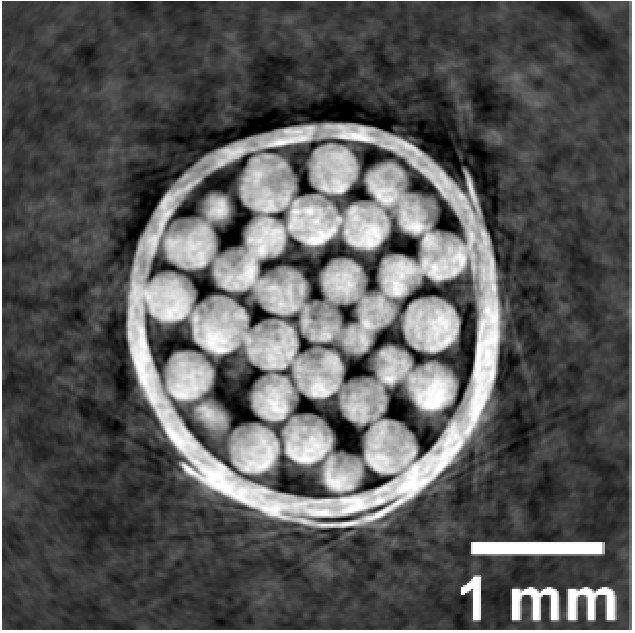
Random-walk tomogram of the plastic spheres phantom. The image is noisier and somewhat less sharp compared to other cycloidal implementations.

While the random-walk scan was primarily applied to demonstrate the knife-edge method’s ability to track arbitrary movements, we have also reconstructed the acquired images and performed a resolution analysis as per the procedure described above. The reconstructed image is shown in Fig. 9, and the resolution was estimated at 34 ± 11 µm. This is slightly worse than the estimates made above for the cycloidal sampling schemes, which is also reflected in the overall image quality. While this may suggest that random sampling may not be an optimal choice (and/or that it requires further investigation, or a different approach to processing the data), our results demonstrate that the tracked motion profile still enables a geometrically accurate tomographic reconstruction.

## 4. Conclusion

In summary, we have discussed a position tracking technique which a) is easy to implement, b) can track the sample motion in cycloidal CT scans thus simplifying and improving the reconstruction process, even for pixelwise acquisitions, and c) provides a testing mechanism for the exploration of entirely new sampling schemes in the future.

We believe these are important findings, as they contribute to the acquisition of high-resolution and high-contrast images of samples within shorter scan times than possible through previous approaches. Moreover, the method’s flexibility and ease of implementation make it a logical component of any setup, cycloidal or otherwise, which requires accurate knowledge of the sample position during a scan. High-resolution scans can particularly benefit from the use of knife-edge tracking, as drifts of the setup are more significant. We are studying the use of masks with increasingly small apertures to flexibly adapt the system’s resolution, and knife-edge tracking will enable us to achieve accurate results even at higher resolution.

Some limitations of the method and, correspondingly, the need for further investigation have emerged. For example, pixelwise cycloidal scans were found to be prone to ring artefacts; to overcome the issue experimentally, rather than by applying software ring artefact removal, scans where the sample “wiggle” is expanded to the width of a few pixels should be implemented.

Other sample tracking approaches are available, and their respective merit will be a subject of further research. While in principle it may be possible to track features other than a sharp edge, we would like to emphasize that, in cycloidal CT, it is paramount that the sample translation is tracked independently from its rotation (for the latter the nominal motor performance can usually be safely assumed). Therefore, fiducial markers attached to the sample itself are not suitable. For the same reason, it may be challenging to track the sample motion through image registration algorithms, as the sample translation and rotation would have to be “separated” from images to which both motions contribute. A laser tracking interferometer which measures the mechanical error in position during a scan could be an alternative to knife-edge tracking. Another realistic (and possibly more robust) alternative would be the use of a motors-encoded readout system synchronized with the detector acquisition, which can output the position of the motors at any given point, such as the ZEBRA implementation at the Diamond Light Source [34,35]. The method in this paper presents an alternative for x-ray systems where this is not available.

## Data Availability

Data underlying the results presented in this paper are not publicly available at this time but may be obtained from the authors upon reasonable request.

## References

[1] Cycloidal Computed Tomography, by Hagen C. K. Vittoria F. Endrizzi M. Olivo A. (2018, Dec 13). UK Patent Application 1820362.0.

[2] Hagen C. K. Vittoria F. A. Roche i Morgo O. Endrizzi M. Olivo A., “Cycloidal computed tomography,” Phys. Rev. Appl. 14(1), 014069 (2020). 10.1103/PhysRevApplied.14.014069 PMC11497279 34169514

[3] Barrie Smith N. Webb A., “General image characteristics, data acquisition and image reconstruction,” in *Introduction to Medical Imaging: Physics* , Engineering and Clinical Applications, ed. (Cambridge University, 2010), Chap. 1, pp. 1-33.

[4] Diemoz P. C. Vittoria F. A. Olivo A., “Spatial resolution of edge illumination X-ray phase-contrast imaging,” Opt. Express 22(13), 15514–15529 (2014). 10.1364/OE.22.015514 24977810

[5] Hagen C. K. Vittoria F. A. Endrizzi M. Olivo A., “Theoretical framework for spatial resolution in edge-illumination x-ray tomography,” Phys. Rev. Appl. 10(5), 054050 (2018). 10.1103/PhysRevApplied.10.054050

[6] Sidky E. Y. Kao C.-M. Pan X., “Accurate image reconstruction from few-views and limited-angle data in divergent-beam CT,” J. X-Ray Sci. Technol. 14, 119–139 (2006). 10.48550/arXiv.0904.4495

[7] Han X. Bian J. Ritman E. L. Sidky E. Y. Pan X., “Optimization-based reconstruction of sparse images from few-view projections,” Phys. Med. Biol. 57(16), 5245–5273 (2012). 10.1088/0031-9155/57/16/5245 22850194 PMC3446871

[8] Hsieh J. Wei Y. Wang G., “Fractional scan algorithms for low-dose perfusion CT,” Med. Phys. 31(5), 1254–1257 (2004). 10.1118/1.1708653 15191317

[9] Anirudh R. Kim H. Thiagarajan J. J. Mohan K. A. Champley K. Bremer T., “Lose the views: Limited angle ct reconstruction via implicit sinogram completion,” in Proceedings of the IEEE Conference on Computer Vision and Pattern Recognition, (2017), pp. 6343–6352.

[10] Pelt D. M. Batenburg J. K. Sethian J. A., “Improving Tomographic Reconstruction from Limited Data Using Mixed-Scale Dense Convolutional Neural Networks,” J. Imaging 4(11), 128 (2018). 10.3390/jimaging4110128

[11] Je U. Cho H. Lee M. Oh J. Park Y. Hong D. Park C. Cho H. Choi S. Koo Y., “Dental cone-beam CT reconstruction from limited-angle view data based on compressed-sensing (CS) theory for fast, low-dose X-ray imaging,” J. Korean Phys. Soc. 64(12), 1907–1911 (2014). 10.3938/jkps.64.1907

[12] Cho S. Lee T. Min J. Chung H., “Feasibility study on many-view under-sampling technique for low-dose computed tomography,” Opt. Eng. 51(8), 080501 (2012). 10.1117/1.OE.51.8.080501

[13] Abbas S. Lee T. Shin S. Lee R. Cho S., “Effects of sparse sampling schemes on image quality in low-dose CT,” Med. Phys. 40(11), 111915 (2013). 10.1118/1.4825096 24320448

[14] Koesters T. Knoll F. Sodickson A. Sodickson D. K. Otazo R., “Sparsect: interrupted-beam acquisition and sparse reconstruction for radiation dose reduction,” Proc. SPIE 10132, 101320Q (2017). 10.1117/12.2255522

[15] Olivo A., “Edge-Illumination x-Ray Phase-Contrast,” J. Phys.: Condens. Matter 33(36), 363002 (2021). 10.1088/1361-648X/ac0e6e PMC8276004 34167096

[16] Vittoria F. Endrizzi M. Diemoz P. Zamir A. Wagner U. H. Rau C. Robinson I. K. Olivo A., “X-ray absorption, phase and dark-field tomography through a beam tracking approach,” Sci. Rep. 5(1), 16318 (2015). 10.1038/srep16318 26541117 PMC4635357

[17] Viermetz M. Birnbacher L. Willner M. Achterhold K. Pheiffer F. Herzen J., “High resolution laboratory grating-based X-ray phase-contrast CT,” Sci. Rep. 8(1), 15884 (2018). 10.1038/s41598-018-33997-5 30367132 PMC6203738

[18] Dreier T. Peruzzi N. Lundström U. Bech M., “Improved resolution in x-ray tomography by super-resolution,” Appl. Opt. 60(20), 5783–5794 (2021). 10.1364/AO.427934 34263797

[19] Massimi L. Hagen C. K. Endrizzi M. Munro P. R. T. Havariyoun G. Hawker P. M. S. Smit B. Astolfo A. Larkin O. J. Waltham R. M. Shah Z. Duggy S. W. Nelan R. L. Peel A. Suaris T. Jones J. L. Haig I. G. Olivo A., “Laboratory-based x-ray phase contrast CT technology for clinical intra-operative specimen imaging,” Proc. SPIE 10948, 109481R (2019). 10.1117/12.2511770

[20] Roche i Morgó O. Massimi L. Suaris T. Endrizzi M. Munro P. R. T. Savvidis S. Havariyoun G. Hawker P. M. S. Astolfo A. Larkin O. J. Nelan R. L. Jones J. L. Pelt D. M. Bate D. Olivo A. Hagen C. K., “Exploring the potential of cycloidal computed tomography for advancing intraoperative specimen imaging,” Proc. SPIE 11840, 118400R (2021). 10.1117/12.2594547

[21] Roche i Morgó O. Vittoria F. Endrizzi M. Olivo A. Hagen C. K., “Technical Note: Practical implementation strategies of cycloidal computed tomography,” Med. Phys. 48(10), 6524–6530 (2021). 10.1002/mp.14821 34169514 PMC11497279

[22] Davis T. J. Gao D. Gureyev T. E. Stevenson A. W. Wilkins S. W., “Phase-contrast imaging of weakly absorbing materials using hard X-rays,” Nature 373(6515), 595–598 (1995). 10.1038/373595a0

[23] Bravin A. Coan P. Suortti P., “X-ray phase-contrast imaging: from pre-clinical applications towards clinics,” Phys. Med. Biol. 58(1), R1–R35 (2013). 10.1088/0031-9155/58/1/R1 23220766

[24] Olivo A. Speller R. D., “A coded-aperture technique allowing x-ray phase contrast imaging with low-brilliance x-ray sources,” Appl. Phys. Lett. 91(7), 074106 (2007). 10.1063/1.2772193

[25] Olivo A. Speller R., “Modelling of a novel x-ray phase contrast imaging technique based on coded apertures,” Phys. Med. Biol. 52(22), 6555–6573 (2007). 10.1088/0031-9155/52/22/001 17975283

[26] Diemoz P. C. Vittoria F. A. Hagen C. K. Endrizzi M. Coan P. Bravin A. Wagner U. H. Rau C. Robinson I. K. Olivo A., “A single-image retrieval method for Edge illumination x-ray phase-contrast imaging: application and noise analysis,” Phys. Med. 32(12), 1759–1764 (2016). 10.1016/j.ejmp.2016.07.093 27836637

[27] Diemoz P. C. Hagen C. K. Endrizzi M. Minuti M. Bellazzini R. Urbani L. De Coppi P. Olivo A., “Single-shot x-ray phase-contrast computed tomography with non-microfocal laboratory sources,” Phys. Rev. Appl. 7(4), 044029 (2017). 10.1103/PhysRevApplied.7.044029

[28] Weitkamp T. Diaz A. David C. Pfeiffer F. Stampanoni M. Cloetens P. Ziegler E., “X-Ray Phase Imaging with a Grating Interferometer,” Opt. Express 13(16), 6296–6304 (2005). 10.1364/OPEX.13.006296 19498642

[29] Wen H. H. Bennett E. E. Kopace R. Stein A. F. Pai V., “Single-shot x-ray differential phase-contrast and diffraction imaging using two-dimensional transmission gratings,” Opt. Lett. 35(12), 1932–1934 (2010). 10.1364/OL.35.001932 20548343 PMC3091831

[30] Rix K. R. Dreier T. Shen T. Bech M., “Super-resolution x-ray phase-constrast and dark-field imaging with a single 2D grating and electromagnetic source stepping,” Phys. Med. Biol. 64(16), 165009 (2019). 10.1088/1361-6560/ab2ff5 31284279

[31] Savvidis S. Gerli M. F. M. Pellegrini M. Massimi L. Hagen C. K. Endrizzi M. Atzeni A. Ogunbiyi O. K. Turmaine M. Smith E. S. Fagiani C. Selmin G. Urbani L. Durkin N. Shibuya S. De Coppi P. Olivo A., “Monitoring tissue engineered constructs and protocols with laboratory-based x-ray phase contrast tomography,” Acta Biomater. 141, 290–299 (2022). 10.1016/j.actbio.2022.01.022 35051630

[32] Münch B. Trtik P. Marone F. Stampanoni M., “Stripe and ring artifact removal with combined wavelet — Fourier filtering,” Opt. Express 17(10), 8567–8591 (2009). 10.1364/OE.17.008567 19434191

[33] Raven C., “Numerical removal of ring artifacts in microtomography,” Rev. Sci. Instrum. 69(8), 2978–2980 (1998). 10.1063/1.1149043

[34] Cobb T. Chernousko Y. Uzun I., “ZEBRA: A flexible solution for controlling scanning experiments,” in Proceedings of ICALEPCS2013 (2013) pp. 739–763.

[35] Batey D. Rau C. Cipiccia S., “High-speed X-ray ptychographic tomography,” Sci. Rep. 12(1), 7846 (2022). 10.1038/s41598-022-11292-8 35551474 PMC9098852

